# The Association of Bread and Rice with Metabolic Factors in Type 2 Diabetic Patients

**DOI:** 10.1371/journal.pone.0167921

**Published:** 2016-12-22

**Authors:** Mahdieh Akhoundan, Zhaleh Shadman, Parisa Jandaghi, Maryam Aboeerad, Bagher Larijani, Zahra Jamshidi, Hamidreza Ardalani, Mohsen Khoshniat Nikoo

**Affiliations:** 1 Diabetes Research Center, Endocrinology and Metabolism Clinical Sciences Institute, Tehran University of Medical Sciences, Tehran, Iran; 2 Endocrinology and Metabolism Research Center, Endocrinology and Metabolism Research Center (EMRC), Endocrinology and Metabolism Research Institute, Tehran University of Medical Sciences, Tehran, Iran; 3 Endocrinology and Metabolism Research Center, Endocrinology and Metabolism Clinical Sciences Institute, Tehran university of Medical Sciences, Tehran, Iran; 4 Department of Horticultural Sciences, Science and Research Branch, Islamic Azad University, Tehran, Iran; Weill Cornell Medical College Qatar, QATAR

## Abstract

**Purpose:**

Carbohydrates are shown to have an important role in blood glucose control, type 2 diabetes and cardiovascular diseases risk. This is even more challenging when considering populations consuming refined grains diets. Bread and rice are staple foods which supply main proportion of Iranian calorie intake. This study was designed to investigate the effect of bread and rice intake on blood glucose control, lipid profile and anthropometric measurements in Iranian type 2 diabetic patients.

**Methods:**

426 patients with type 2 diabetes were included in this study. Anthropometric measurements were done using standard methods. Dietary information was assessed by a valid and reliable food frequency questionnaire (FFQ). Fasting blood glucose (FBG), glycated hemoglobin (HbA1c), serum triglycride (TG), total cholesterol (TC), low density lipoprotein (LDL) and high density lipoprotein (HDL) cholesterol were examined after 12-hour fasting.

**Results:**

The results represented that people in the highest tertile compared to the lowest tertile of calorie adjusted total bread intake have higher FBG. FBG in the highest tertile of calorie adjusted total bread-rice intake was also significantly higher than the lowest. The association remained significant after adjusting for potential confounders. Rice intake showed no association with cardio-metabolic risk factors.

**Conclusion:**

We founded that higher total bread intake and total bread-rice intake were associated with FBG in type 2 diabetic patients whereas rice intake was not associated with glucose and lipid profile. This result should be confirmed in prospective studies, considering varieties, glycemic index (GI), glycemic load (GL) and cooking method of bread and rice.

## Introduction

One of the most important chronic disease seems to be Type 2 diabetes [1] that is rapidly increasing throughout the world [2]. Previous studies have shown that 6% of the world's adult population is affected by this disease [3]. According to the World Health Organization (WHO) by 2025, approximately 2.5 million Iranians will suffer from diabetes [4]. Prevalence of type 2 diabetes in Asia, Middle East and Iran have been reported 1.2–14.6%, 4.6–40% and 1.3–14.5%, respectively [5]. The disease is associated with several macro and micro vascular complications, including cardiovascular disease (CVD), retinopathy, neuropathy and nephropathy. A remarkable increase in the prevalence of diabetes, the related costs and difficulties caused by diabetes and its complications are emerging as public health concerns [6]. Dietary intake has been proposed as a modifiable risk factor which is related to type 2 diabetes both directly and indirectly [7]. Studies have shown that type and quantity of dietary carbohydrates play a crucial role in the development of impaired glucose and lipid profile, insulin resistance and increase the risk of type 2 diabetes and CVD [7]. Refined-grains containing low fiber and rich in carbohydrates [8] have a high GL and are quickly absorbed [9]. Studies have demonstrated diets with high GI can increase postprandial blood glucose level and insulin demand and may cause hypertension, dyslipidemia, and insulin resistance in the long term [10]. Comparing the effect of refined grains with whole grains on the risk of type 2 diabetes, studies in the United States have shown that consumption of white rice (≥5 serving per week versus <1 serving per month) [11] and refined grains [12] augmented the risk of type 2 diabetes. The risk of diabetes and metabolic syndrome in Asian populations for whom white rice is their staple food has been estimated high [13]. Studies conducted in Japan showed that white rice consumption is associated with increased risk of type 2 diabetes and metabolic syndrome [14]. In a study in China, high intake of refined grains (rice, bread and noodles) was significantly linked to increased risk of type 2 diabetes [15]. In a cross-sectional study on Indian people who provide almost half of the daily calorie intake from refined grains (including refined wheat, white rice and semolina flour), refined grain intake was significantly associated with high FBG [16].

Nationwide household consumption survey indicated that plant foods have a considerable proportion in Iranian diet. Dietary carbohydrate and fat provide almost 66% and 22% of Iranian daily calorie intake respectively [17], and bread and rice are major amounts of carbohydrate (49%) [18].

Few studies have examined the effect of refined grains intake on glycemic control in diabetic patients [19]. In a study conducted in Iran, on type 2 diabetic patients, Western dietary pattern characterized by high intake of refined grains was significantly associated with the increase of FBG [20]. Finally, this study aimed to investigate the effect of bread and rice intake on blood glucose, lipid profile and anthropometric measurements in type 2 diabetic patients.

## Materials and Methods

This study was approved by the Ethics Committee of Endocrinology and Metabolism Research Center (ethics code E00192) (S1 Fig). The study was conducted on type 2 diabetic patients attending the diabetes and metabolic diseases clinic. In this cross-sectional study 426 patients with type 2 diabetes were recruited by convenience sampling. According to the inclusion criteria, patients aged 35–60 years who were willing to cooperate, were diagnosed with diabetes for more than 5 years, not pregnant, had no history of angina, myocardial infarction or stroke within the last year, did not suffer renal disease or severe liver, thyroid, chronic inflammatory diseases and chronic and severe diabetes complications and not treated with insulin were entered in our study. Exclusion criteria was failure to respond to more than 20% (>34) of all food items in FFQ. After describing the study objectives and design, written informed consent was obtained from all participants. General information including age, sex, duration of diabetes, weight, height and waist circumference were collected. Usual dietary intakes during the last year was assessed using a valid and reliable FFQ [21]. Then, applying household measures [22], reported dietary intakes were converted to grams of food consumed per day for each person. Physical activity levels (PALs) were obtained by calculating the metabolic equivalent (MET) through a validated physical activity questionnaire [23]. Height and weight were measured (to the nearest 0.1 cm and 0.1 kg) using Seca wall meter and Seca digital scale, respectively. Waist circumference was measured via placing the tape between the last rib and the iliac bone (Seca Corporation, Chino, California, USA). FBG, HbA1c, TG, total cholesterol, LDL and HDL cholesterol were measured in venous blood samples obtained after 12-hour fasting.

Serum was immediately separated by centrifuging samples at 3000 rpm at the room temperature for 10 minutes and stored at minus 80 degrees until analysis. Plasma glucose concentration was measured by fluorometric method according glucose oxidase principle (Glucose determination kit, Parsazmun, Tehran, Iran) through auto-analyzer instrument (Hitachi 902, Roche, Basel, Switzerland). Glycated hemoglobin was determined on whole blood sample by HbA1c Pink Kit and DS5 analyzer. The intra assay coefficient of variation (CV %) for glucose and HbA1c were 1.4% and 3.7%, and the inter assay coefficient of variation for them were 1.9% and 3.5%, respectively. Serum TG, TC, LDL and HDL cholesterol were measured by the related biochemical kits (Parsazmun, Tehran, Iran), using the auto-analyzer (Hitachi 902, Roche, Basel, Switzerland). The intra assay CV% for the lipids were 4.1%, 1.3%, 2.0% and 1.8% and the inter assay CV% were 4.5%, 2.0%, 2.3%, and 2.05 respectively. Before start, this study was reviewed and approved by Endocrinology and Metabolism Research Institute, Tehran University of Medical Sciences, Tehran, Iran committee with code of ethics E-00192.

### Statistical analysis

All statistical analyses were performed by SPSS software (version 20.0; SPSS Inc, Chicago) and a p-value < 0.05 showed statistical significance. Normality of the variables was assessed by Kolmogorov–Smirnov test. General characteristics were presented as mean (standard deviation) and in tertile distribution. Analysis of variance was used to compare variables in tertiles of energy-adjusted total bread, rice and total bread-rice intakes with a subsequent Bonferroni correction test. At first, analysis of variance and crude linear regression were used to compare variables in tertiles of bread and rice intakes to find possible associated confounders. In the next step, multivariate linear regression model as related confounders adjusted linear regression models were used to assess the association of metabolic risk factors by tertiles of energy adjusted total bread, rice and also, total rice-bread intake.

## Results

426 individuals with a mean age of 54.4± 7.9 years participated in the study. 82 percent of them were female and 33.7% had a higher than high school education. Some demographic and anthropometric data of the participants mean daily dietary intake and percentile distribution of calorie, bread, rice, some food groups and food items were presented in Table 1. The distribution of characteristics, dietary items and metabolic profiles across tertiles of calorie-adjusted total bread, rice and total bread-rice intake were presented in Tables 2–4. Patients in the highest tertile of calorie-adjusted total bread intake had a lower consumption of fruits (b = -0.26, *P*<0.001), vegetables (b = -0.14, *P* = 0.02) and meat (b = -0.14, *P* = 0.02) and were less likely to consume fiber (b = -0.25, *P*<0.001) Compared with those who are in the lowest tertile. No significant difference was observed in anthropometric and metabolic profile (Table 2). Table 3 showed that the intake of fruits and fiber were decreased by tertiles of calorie-adjusted rice intake (b = -0.22, *P* = 0.001 and b = -0.14, *P* = 0.02) respectively. The people who had higher intake of calorie-adjusted total bread-rice were likely to be younger (Table 4). Intake of Fruits, vegetables and fiber were negatively associated with tertiles of calorie-adjusted total bread-rice intake (Table 4).

**Table 1:** General characteristics, mean daily intake and percentile distribution of energy, some food groups and food items

Characteristics		P1	P2
**Sex (%)**			
**Men**	78(18.3)		
**Women**	348 (81.7)		
**Education (%)**			
**Illiterate**	61 (14.3)		
**Primary**	117 (27.5)		
**Guidance school**	67 (15.7)		
**High school**	131 (30.8)		
**University**	50 (11.7)		
**Smoking status (%)**			
**Current**	16 (3.8)		
**Never**	383 (89.9)		
**Ex-smoker**	27 (6.3)		
**Age (yr)**	54.4 ± 7.9	51.00	60.00
**BMI (kg/m**^**2**^**)**	29.4 ± 5.1	26.37	30.12
**Waist circumference (cm)**	96.7 ± 12.4	90.00	100.33
**PAL (MET)**	1.4 ± 0.2	1.37	1.53
**Kcal/d**	2236.7 ± 746.9	1709	2663
**Bread (g/d)**	152.6 ± 126.3	78.32	168.08
**Rice (g/d)**	172.3 ± 116.7	107.00	250.00
**Total bread and rice (g/d)**	324.9 ±162.1	280.5	421.8
**Bread (g/1000 kcal)**	65.7 ± 40.3	42.55	75.25
**Rice (g/1000 kcal)**	81.7 ± 58.6	50.51	94.15
**Total bread and rice (g/1000 kcal)**	147.5 ± 63.3	117.19	169.31
**Food groups:**			
**Fruits (g/d)**	653.9 ± 414.1	278.3	1006.6
**Vegetables (g/d)**	414.6 ± 218.2	300.9	543.1
**Dairy (g/d)**	463.8 ± 286.8	315.6	601.2
**Meat (g/d)**	86.7 ± 42.2	63.4	111.0
**Fiber (g/d)**	30.5 ± 13.6	22.10	29.5

Data are presented asmean ± SD or n (%)

P1 and P2 are the levels of percentiles 33.33% and 66.66%, respectively

**Table 2:** Characteristics, dietary and cardiometabolic variables in tertiles of energy-adjusted total bread intake

Characteristics	Tertiles of energy-adjusted total bread intake*			Beta	*P*^†^	P**
	1	2	3			
	<42.55	42.55–75.25	>75.25			
**Age (yr)**	53.2±7.6	55.9±7.7	54.1±8.2	0.04	0.44	0.08
**BMI (kg/m**^**2**^**)**	29.7±5.1	28.8±4.9	29.4±5.1	-0.001	0.98	0.54
**Waist circumference (cm)**	96.1±11.6	96.7±11.6	97.3±14.0	0.03	0.59	0.86
**PAL (MET)**	1.5±0.1	1.5±0.2	1.4±0.2	-0.09	0.14	0.14
**Dairy (g/d)**	224.1±115.6	223.3±114.0	197.1±117.7	-0.09	0.14	0.24
**Fruits (g/d)**	326.4±148.6^‡^	282.9±124.3	243.5±99.6^‡^	-0.26	<0.001	<0.001
**Vegetables (g/d)**	218.0±118.7	183.7±71.7	182.8±104.0	-0.14	0.02	0.04
**Meat (g/d)**	43.2±18.3	40.1±14.9	36.9±20.5	-0.14	0.02	0.09
**Fiber (g/d)**	14.9±3.7^‡^	13.2±3.3^‡^	12.7±2.7^‡^	-0.25	<0.001	<0.001
**FBG (mg/dl)**	170.3±66.1	172.2±66.8	169.7±66.6	-0.004	0.95	0.96
**HbA1c (mg/dl)**	7.6±2.2	7.3±1.8	7.4±1.7	-0.04	0.49	0.56
**TG (mg/dl)**	152.4±80.9	174.8±112.1	163.1±96.1	0.04	0.49	0.34
**Total cholesterol (mg/dl)**	164.9±39.6	164.5±40.8	168.5±46.4	0.03	0.59	0.8
**LDL—cholesterol (mg/dl)**	89.3±27.1	87.7±22.8	90.4±28.5	0.01	0.79	0.79
**HDL- cholesterol (mg/dl)**	49.3±11.4	54.5±69.9	48.7±12.8	-0.007	0.91	0.62

*Quantitative data presented as the percent and mean ± SD in tertiles

**P-value of analysis of variance for quantitative variables

^†^ *P* -value of linear regression

^‡^ Bonferroni test showed a significant difference between T_1_—T_3_ and T_2_—T_3_

**Table 3:** Characteristics, dietary and cardiometabolic variables in tertiles of energy-adjusted rice intake

Characteristics	Tertiles of energy-adjusted rice intake*			Beta	*P*^†^	P**
	1	2	3			
	<50.51	50.51–94.15	>94.15			
**Age (yr)**	55.1±7.5	54.8±7.7	53.3±8.4	-0.09	0.16	0.33
**BMI (kg/m**^**2**^**)**	29.7±4.9	29.4±5.1	29.3±5.4	-0.05	0.41	0.71
**Waist circumference (cm)**	97.1±13.9	97.1±10.9	96.1±12.1	-0.03	0.60	0.84
**PAL (MET)**	1.4±0.2	1.4±0.1	1.5±0.2	0.01	0.79	0.80
**Dairy (g/d)**	217.1±113.5	215.1±122.9	212.5±112.5	-0.01	0.80	0.91
**Fruits (g/d)**	316.4±154.6^‡^	290.1±110.9	246.2±110.5^‡^	-0.22	0.001	0.002
**Vegetables (g/d)**	196.3±116.4	205.6±104.9	182.3±77.1	-0.05	0.38	0.33
**Meat (g/d)**	36.4±16.5^‡^	43.9±23.2^‡^	39.7±12.4	0.07	0.25	0.03
**Fiber (g/d)**	14.1±3.3^‡^	13.8±3.7	12.9±3.1^‡^	-0.14	0.02	0.06
**FBG (mg/dl)**	116.1±67.3	171.5±69.4	174.6±62.2	0.05	0.41	0.71
**HbA1c (mg/dl)**	7.3±1.6	7.6±2.1	7.4±2.1	0.01	0.81	0.77
**TG (mg/dl)**	166.3±95.8	171.4±110.4	152.6±83.9	-0.05	0.37	0.45
**Total cholesterol (mg/dl)**	160.5±40.9	173.9±43.8	163.5±41.3	0.02	0.66	0.10
**LDL—cholesterol (mg/dl)**	85.3±24.6	93.1±25.76	89.11±27.7	0.05	0.36	0.17
**HDL- cholesterol (mg/dl)**	57.1±70.4	47.3±10.8	48.2±11.2	-0.08	0.18	0.26

*Quantitative data presented as the percent and mean ± SD in tertiles

**P-value of analysis of variance for quantitative variables

^†^ *P* -value of linear regression

^‡^ Bonferroni test showed a significant difference between T_1_—T_3_ and T_2_—T_3_

**Table 4:** Characteristics, dietary and cardiometabolic variables in tertiles of energy-adjusted total bread-rice intake

Characteristics	Tertiles of energy-adjusted total bread-rice intake*			Beta	*P*^†^	P**
	1	2	3			
	<117.19	117.19–169.31	>169.31			
**Age (yr)**	54.9±6.9	55.8±8.6^‡^	52.5±7.9^‡^	-0.12	0.06	0.02
**BMI (kg/m**^**2**^**)**	29.4±4.7	28.9±4.9	29.8±5.6	0.03	0.65	0.58
**Waist circumference (cm)**	95.9±12.2	96.8±12.5	97.5±12.5	0.05	0.45	0.75
**PAL (MET)**	1.5±0.1	1.4±0.2	1.4±0.2	-0.08	0.21	0.44
**Dairy (g/d)**	218.5±110.5	232.2±104.7	193.4±129.5	-0.08	0.17	0.09
**Fruits (g/d)**	344.3±153.5^‡^	286.1±100.4^‡^	223.3±100.7^‡^	-0.38	<0.001	<0.001
**Vegetables (g/d)**	210.1±115.9	197.6±88.7	176.9±95.1	-0.13	0.03	0.11
**Meat (g/d)**	41.9±20.2	40.4±13.8	37.8±19.9	-0.09	0.16	0.37
**Fiber (g/d)**	15.1±4.9^‡^	13.6±3.1^‡^	12.3±2.3^‡^	-0.32	<0.001	<0.001
**FBG (mg/dl)**	162.1±64.3	169.5±63.4	180.6±70.3	0.11	0.07	0.21
**HbA1c (mg/dl)**	7.4±2.1	7.5±1.8	7.5±1.9	0.3	0.64	0.89
**TG (mg/dl)**	158.5±91.2	174.5±119.2	157.1±76.1	-0.006	0.92	0.45
**Total cholesterol (mg/dl)**	158.7±35.8	173.1±50.1	166.1±38.5	0.07	0.27	0.10
**LDL—cholesterol (mg/dl)**	84.7±22.2	91.5±28.5	91.2±26.8	0.102	0.11	0.17
**HDL- cholesterol (mg/dl)**	55.5±70.7	48.4±11.7	48.8±12.7	-0.06	0.31	0.48

*Quantitative data presented as the percent and mean ± SD in tertiles

**P-value of analysis of variance for quantitative variables

^†^ *P* -value of linear regression

^‡^ Bonferroni test showed a significant difference between T_1_—T_3_ and T_2_—T_3_

Tables 5–7 present the metabolic abnormalities according to the tertiles of total bread, rice and total bread-rice intake, respectively. After adjusting for age and sex, we observed a significant association between calorie-adjusted total bread intake and FBG (*P* = 0.03). After additional adjustment for fruit, vegetables and fiber intake and also duration of diabetes, the association remained significant (*P* = 0.04).

**Table 5:** Multivariate adjusted association of metabolic risk factors by tertiles of energy adjusted total bread intake

	Model 1		Model 2		Model 3		Model 4	
	Beta (SE)	P value	Beta (SE)	P value	Beta (SE)	P value	Beta (SE)	P value
**FBG**	0.32(5.27)	0.03	0.31 (5.74)	0.03	0.28 (5.73)	0.04	0.27(5.73)	0.04
**HbA1c**	0.09 (0.15)	0.12	0.07 (0.16)	0.20	0.06 (0.16)	0.25	0.04 (0.16)	0.31
**TG**	0.002(7.80)	0.98	0.08 (0.001)	0.17	0.07(0.001)	0.21	0.07(0.001)	0.21
**TC**	0.08(0.001)	0.22	0.07(0.001)	0.22	0.07(0.001)	0.26	0.06(0.001)	0.32
**HDL-c**	0.01(0.001)	0.82	0.01(0.001)	0.76	0.02(0.001)	0.76	0.02(0.001)	0.71
**LDL-c**	0.05(0.002)	0.44	0.04(0.002)	0.51	0.03(0.002)	0.56	0.03(0.002)	0.64

FBG fasting blood glucose, HbA1c hemoglobin A1c, TG triglyceride, TC total cholesterol, HDL-c high density lipoprotein, LDL-c low density lipoprotein

In all cases linear regression coefficient was expressed as standardized coefficients Beta and standard error of mean as SE and p value of regression

Model 1 was adjusted for age, sex

Model 2 was adjusted for age, sex, fruit and vegetables intake (g/1000 Kcal per day)

Model 3 was adjusted for age, sex, fruit and vegetables intake (g/1000 Kcal per day), fiber intake (g/1000 Kcal per day)

Model 4 was adjusted for age, sex, fruit and vegetables intake (g/1000 Kcal per day), fiber intake (g/1000 Kcal per day) and duration of diabetes

**Table 6:** Multivariate adjusted association of metabolic risk factors by tertiles of energy adjusted rice intake

	Model 1		Model 2		Model 3		Model 4	
	Beta (SE)	P value	Beta (SE)	P value	Beta (SE)	P value	Beta (SE)	P value
**FBG**	0.05(0.001)	0.43	0.05(5.40)	0.45	0.04(5.33)	0.55	0.04(5.16)	0.45
**HbA1c**	0.01(0.02)	0.82	0.03(0.15)	0.64	0.02(0.15)	0.68	0.008(0.15)	0.80
**TG**	-0.05(7.73)	0.40	-0.04(7.91)	0.54	-0.04(7.90)	0.49	-0.05(8.08)	0.41
**TC**	0.03(3.26)	0.59	0.05(3.34)	0.45	0.04(3.33)	0.50	0.03(3.39)	0.64
**HDL-c**	-0.07(3.27)	0.23	-0.08(3.37)	0.22	-0.08(3.38)	0.32	-0.09(3.47)	0.18
**LDL-c**	0.06(0.002)	0.37	0.07(2.09)	0.28	0.06(2.09)	0.31	0.05(2.13)	0.44

FBG fasting blood glucose, HbA1c hemoglobin A1c, TG triglyceride, TC total cholesterol, HDL-c high density lipoprotein, LDL-c low density lipoprotein

In all cases linear regression coefficient was expressed as standardized coefficients Beta and standard error of mean as SE and p value of regression

Model 1 was adjusted for Age, sex

Model 2 was adjusted for Age, sex, fruit and meat intake (g/1000 Kcal per day)

Model 3 was adjusted for Age, sex, fruit and meat intake (g/1000 Kcal per day), fiber intake (g/1000 Kcal per day)

Model 4 was adjusted for Age, sex, fruit and meat intake (g/1000 Kcal per day), fiber intake (g/1000 Kcal per day) and duration of diabetes

**Table 7:** Multivariate adjusted association of metabolic risk factors by tertiles of energy adjusted total bread-rice intake

	Model 1		Model 2		Model 3		Model 4	
	Beta (SE)	P value	Beta (SE)	P value	Beta (SE)	P value	Beta (SE)	P value
**FBG**	0.24(0.02)	0.03	0.21(0.02)	0.04	0.20(0.02)	0.04	0.19(0.01)	0.04
**HbA1c**	0.03(0.02)	0.63	0.06(0.02)	0.27	0.05(0.02)	0.36	0.04(0.02)	0.53
**TG**	0.002(0.001)	0.98	0.03(0.001)	0.54	0.02(0.001)	0.65	0.01(0.001)	0.76
**TC**	0.08(0.001)	0.18	0.08(0.001)	0.14	0.08(0.001)	0.19	0.06(0.007)	0.33
**HDL-c**	-0.05(0.001)	0.44	-0.04(0.001)	0.44	-0.45(0.001)	0.45	0.09(0.002)	0.11
**LDL-c**	0.10(0.002)	0.10	0.10(0.002)	0.09	0.09(0.002)	0.11	0.07(0.002)	0.21

FBG fasting blood glucose, HbA1c hemoglobin A1c, TG triglyceride, TC total cholesterol, HDL-c high density lipoprotein, LDL-c low density lipoprotein

In all cases linear regression coefficient was expressed as standardized coefficients Beta and standard error of mean as SE and p value of regression

Model 1 was adjusted for Age, sex

Model 2 was adjusted for Age, sex, fruit and vegetable intake (g/1000 Kcal per day)

Model 3 was adjusted for Age, sex, fruit and vegetable intake (g/1000 Kcal per day), fiber intake (g/1000 Kcal per day)

Model 4 was adjusted for Age, sex, fruit and vegetable intake (g/1000 Kcal per day), fiber intake (g/1000 Kcal per day) and duration of diabetes

There was no relation between glucose and lipid profile and calorie adjusted rice intake.

In model 1, subjects in the highest tertile of total bread-rice intake had significantly higher FBG values than those in the lowest tertile (*P* = 0.03). After multivariate adjustment for other confounders (model 4), the association was significant (*P* = 0.04). However, there was no longer association between total bread-rice intake and HbA1c and lipid profile.

## Discussion

In this cross-sectional study on type 2 diabetic patients, higher consumption of total bread was related to increase of the level of FBG. No significant association was observed between glycemic control and lipid profile and tertiles of rice intake. Total bread-rice intake also showed positive relation with FBG. The association remained significant after adjusting for age, sex, fruit and vegetable intake (g/1000 Kcal per day), fiber intake (g/1000 Kcal per day) and duration of diabetes.

It has been demonstrated that the whole grains reduce the risk of diabetes, CVD [24], and metabolic syndrome [25]. However, there are some inconsistencies in the literature investigating refined grains. Some studies have demonstrated the increment [26], no association [24] or even reduction [24] of diabetes and CVD risk and glycemic control. Most of studies regarding carbohydrate consumption have been conducted in Western countries, while in Asian populations carbohydrates constitute higher proportion of daily calorie and the average consumption of refined grains [27], particularly bread and rice are much higher compared to Western countries: 333 g/d in India [16], 201 g/d in Iran [28], 300 g/d in China [27] vs. 117 g/d in Western populations [25].

Our current results were in agreement with previous studies [25] as a higher intake of total bread and total bread-rice was associated with FBG. In the other study evaluating the relation of refined grains intake with the metabolic syndrome (MS) in an urban south Indian population reported that higher refined grain intake was significantly associated with FBG [16]. The results showed that higher intake of refined grain was positively relevant to higher fasting glucose concentrations [25]. But this relation was not confirmed in the Framingham Cohort Study [29]. In fact in this cohort the intake of refined-grain was not associated with concentrations of fasting insulin. On the other hand, some studies in line with our results, reported no association between white rice intake and blood glucose and metabolic risk factors for cardiovascular disease in different populations [24]. Investigating the effects of three popular carbohydrate foods (bread, roti and rice) on increasing triglyceride levels in type 2 diabetic and healthy Indian people showed the rice had the lowest effect on triglycerides increase compared with two other carbohydrate foods [19]. In another study on 3006 non-diabetic Iranian men, no significant association between white rice consumption and FBG and other CVD risk factors was observed [10]. In the other study, after 10 years following-up of 8000 Japanese men reported that men who developed coronary heart disease (CHD) had a significantly lower intake of carbohydrate (mainly supplied from rice) compared with those who did not experience the disease [30]. In another prospective study in Japan, calorie-adjusted rice intake demonstrated an inverse association with CHD, heart failure, and total CVD in men but not in women [31]. In our study no significant difference was observed between diabetic men and women (data not shown). However, some studies demonstrated contradictory results [14].

The amount and quality of dietary carbohydrate affect glycemic and insulin response and lipid profile [24]. The whole grains’s protective action against chronic diseases and the beneficial effects on blood glucose and lipid profile mainly attributed to low glycemic index, high content of fiber [32], vitamins (such as vitamin E), minerals (such as magnesium) [33], phenolic compounds, phytoestrogens which are found in the bran and fibrous components. Moreover, in a cohort study it was found insoluble cereal fiber constitute in whole-grain compared with soluble fiber was the major fiber having a favorable effect on fasting insulin and also Magnesium available in whole-grain could have insulin-sensitizing effect [24]. The components are lost to a large extent during refining process, leading to calorie dense nutritionally poor refined grains with higher glycemic index [34]. The grains particle size, especially consumption of intact form of grains also suggested that may have impact on glycemic response [35].

According to Iranian food consumption pattern, carbohydrates are the main source of daily calorie intake [17] and bread and rice are accounted staple foods of Iranian people and constitute the greatest contribution of calorie intake, respectively.

Despite the benefits of whole grains, the breads that are mostly consumed by Iranian people, are prepared with refined flour [36]. CASPIAN national survey reported that more than 57% of Iranian families consumed refined flour breads [36]. The breads mainly classified as high-glycemic index foods which can explain the relationship between the consumption of bread, and FBG observed in our study. Examining the effects of 4 Iranian traditional breads on blood glucose in type 2 diabetic patients revealed a significant increase in mean blood glucose at 60, 90, 120 and 180 min. These changes were greater for the bread prepared from refined grains [37]. Inconsistency in the effect of bread and rice in our study rather than Western and other Asian studies may be due to differences in lifestyle, eating habits and physical activity [16]. On the other hand, studies have reported glycemic response is influenced by various factors including amount, source, physical and chemical characteristics of the foods and food processing methods. Rice is one of the foods with a wide range of GI values attributed to inherent botanical differences. Amylose content is also a determinant in ability to digest and absorb carbohydrates, the greater amylose content and the lower glycemic response. It is recommended that rice glycemic index be determined brand by brand locally [38]. Another explanation for differences in glycemic responses to rice could be food structure such as particle size and the method of cooking rice [10]. Investigation on glycemic index of some varieties of white rice consumed in Iran, classified the rice as low to medium GI food [39] against the results of other studies such as Filipino indigenous rice cultivars [40] and rice consumed in Japan [41], China [42] and Australia [43].

There are limitations in our study that should be noticed when interpreting the results. This cross-sectional design of the study does not allow causal conclusions. Further, glycemic index of bread and rice is not calculated in this study. A comprehensive table of GI values of Iranian foods is not available.

## Conclusion

We found that higher intake of total bread and total bread-rice were associated with FBG in type 2 diabetic patients. The association remained significant after adjusting for possible confounders. However, no association was detected between white rice intake and cardio-metabolic risk factors. This result can be attributed to the type, GI and GL and cooking method of rice in our population. It seems necessary to design prospective study to assess the effect of bread and rice intake on cardio-metabolic risk factor in long time, emphasizing on the varieties, physico-chemical characteristics and processing and cooking method of bread and rice.

## Supporting Information

S1 Fig Ethical clearance approval. The approval certificate.
(JPG)
