# Supplementary figures and images for: The Association of Bread and Rice with Metabolic Factors in Type 2 Diabetic Patients
Source: PLoS One. 2016 Dec 22;11(12):e0167921. doi: 10.1371/journal.pone.0167921 (PMC5179013; doi:10.1371/journal.pone.0167921)

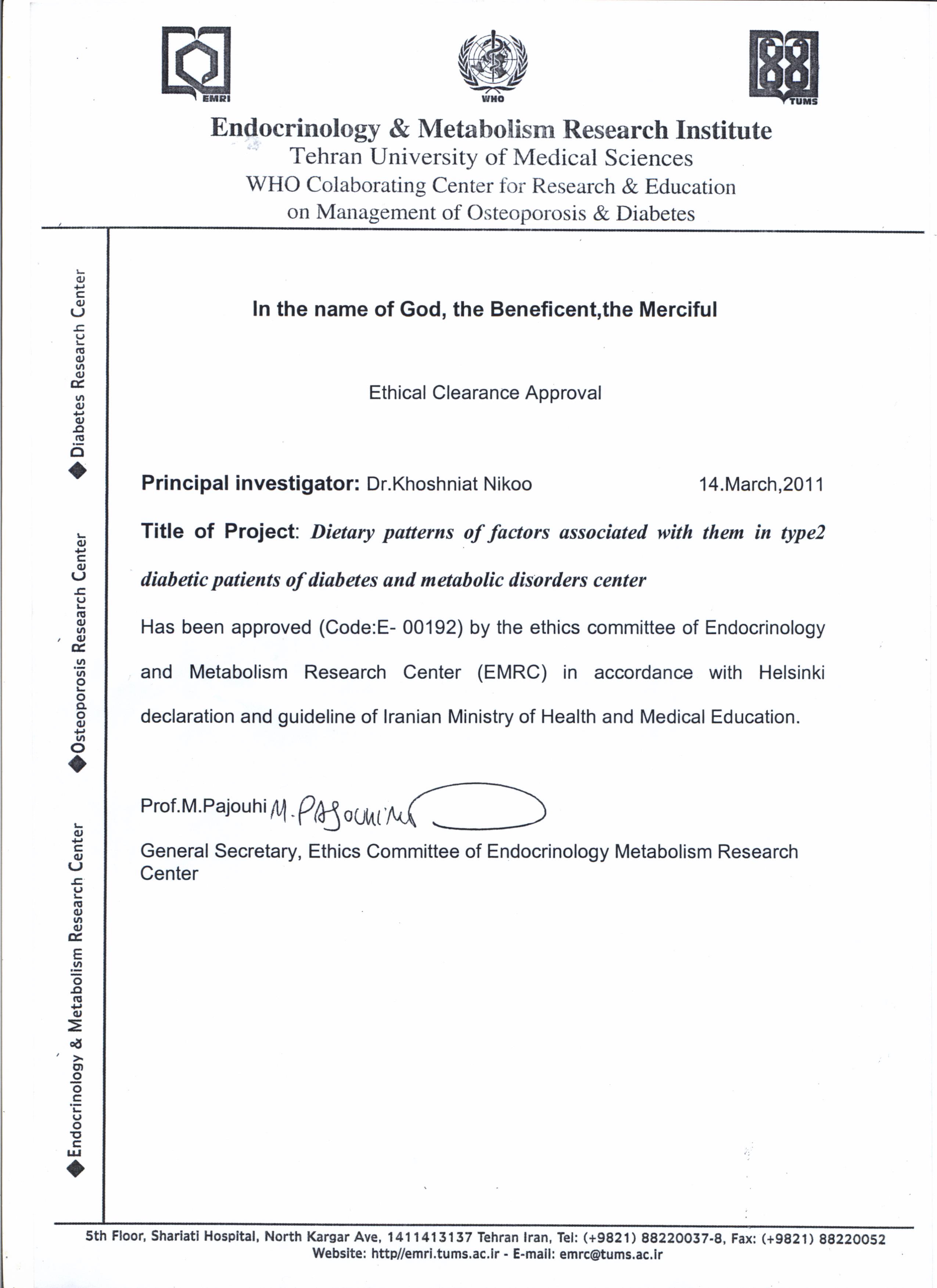

Supplement: S1 Fig — The approval certificate. (JPG) [file pone.0167921.s001.jpg]
